# Acute non-alcoholic caffeinated beverage consumption as a trigger for cryptogenic ischemic stroke in the young: findings from the SECRETO study

**DOI:** 10.1007/s00415-026-13882-2

**Published:** 2026-06-11

**Authors:** Phillip Ferdinand, Ram Bajpai, Bettina von Sarnowski, Pauli Ylikotila, Marialuisa Zedde, Tomi Sarkanen, Kristina Ryliskiene, Nicolas Martinez-Majander, Lauri Tulkki, Radim Licenik, Janika Kõrv, Liisa Kõrv, Alessandro Pezzini, Ana Catarina Fonseca, Patricia Martinez-Sanchez, Laura Amaya-Pascasio, Nilufer Yesilot, Ulrike Waje-Andreassen, Annette Fromm, Juha Huhtakangas, Ulla Junttola, Pekka Jäkälä, Anne-Mari Kantanen, Petra Redfors, Juha Sinisalo, Eva Gerdts, Turgut Tatlisumak, Christine Roffe, Jukka Putaala

**Affiliations:** 1 Neurosciences, University Hospitals of North Midlands NHS Trust, Stoke-on-Trent, UK; 2 School of Medicine, Keele University, Staffordshire, UK; 3 Department of Neurology, University Medicine Greifswald, Greifswald, Germany; 4 Department of Neurology, Turku University Hospital and University of Turku, Turku, Finland; 5 Neurology Unit, Stroke Unit, Azienda Unità Sanitaria Locale-IRCCS Reggio Emilia, Reggio Emilia, Italy; 6 Department of Neurology, Tampere University Hospital, Tampere, Finland; 7 Faculty of Medicine and Health Technology, Tampere University, Tampere, Finland; 8 Faculty of Medicine, Center of Neurology, Vilnius University, Vilnius, Lithuania; 9 Department of Neurology, Helsinki University Hospital and University of Helsinki, Helsinki, Finland; 10 North West Anglia NHS Foundation Trust Acute Stroke Centre, Peterborough, UK; 11 Department of Neurology and Neurosurgery, University of Tartu, Tartu, Estonia; 12 Department of Medicine and Surgery, University of Parma, Parma, Italy; 13 Stroke Care Program, Department of Emergency, Parma University Hospital, Parma, Italy; 14 Hospital Santa Maria, CHLN, Faculty of Medicine, University of Lisbon, Lisbon, Portugal; 15 Department of Neurology, Torrecardenas University Hospital, University of Almería, Almería, Spain; 16 Istanbul Faculty of Medicine, Department of Neurology, Istanbul University, Istanbul, Turkey; 17 Department of Neurology, Haukeland University Hospital, Bergen, Norway; 18 Department of Neurology, Oulu University Hospital and University of Oulu, Oulu, Finland; 19 Neurocenter Neurology, Kuopio University Hospital, Kuopio, Finland; 20 University of Eastern Finland, Joensuu, Finland; 21 Department of Neurology, Sahlgrenska University Hospital, Gothenburg, Sweden; 22 Department of Clinical Neuroscience, Institute of Neuroscience and Physiology, Sahlgrenska Academy at University of Gothenburg, Gothenburg, Sweden; 23 Department of Cardiology, Heart and Lung Center, Helsinki University Hospital and University of Helsinki, Helsinki, Finland; 24 Center for Research On Cardiac Disease in Women, Department of Clinical Science, University of Bergen, Bergen, Norway

**Keywords:** Young ischemic stroke, Cryptogenic ischemic stroke, Triggers of ischemic stroke, Case-crossover analysis

## Abstract

**Introduction:**

Cryptogenic ischemic stroke (CIS) is responsible for the increase in young ischemic strokes. We investigated non-alcoholic caffeinated beverages as a trigger for young CIS, in a large multi-center, case–control study.

**Methods:**

In Searching for Explanations for Cryptogenic Stroke in the Young: Revealing the Etiology, Triggers, and Outcome (SECRETO; NCT01934725), patients 18–49 years old suffering first ever CIS were recruited within 2 weeks of symptom onset. A structured questionnaire obtained information on coffee, tea, and cola consumption in the last 12 months, usual daily consumption, consumption 24 h preceding stroke and timing of the last consumption prior to stroke. Case-crossover analysis was performed using the Mantel–Haenszel method.

**Results:**

598 CIS patients (54.7% male, mean age 39.4 [standard deviation 8.2]) were analyzed. Case-crossover analysis found association with coffee consumption in the 1-h (relative risk [RR] 1.73, 95% confidence interval [CI] 1.06–2.83) and 2-h (RR 2.15, 95%CI 1.40–3.29) hazard periods, tea consumption in the 1-h (RR 3.93, 95%CI 1.04–14.95) and 2- hour (RR 4.89, 95%CI 1.92–12.50) hazard periods and cola consumption (RR 3.70, 95%CI 1.18–11.62) in the 2-h hazard period. Sub-group analysis in the 2-h hazard period found coffee consumption was associated in both sexes and age groups, high-risk patent foramen ovale (PFO), mild/moderate stress and both low-/high-risk factor burden. Tea consumption was associated in both sexes and age groups and those without PFO.

**Conclusion:**

Coffee, tea, and cola consumption as potential trigger factors were associated with young CIS patients within the 2-h hazard period, with coffee and tea maintaining association in several sub-groups.

**Supplementary Information:**

The online version contains supplementary material available at 10.1007/s00415-026-13882-2.

## Introduction

The burden of ischemic stroke in the young is increasing [1]. Data from a long-standing United Kingdom cohort have shown that the increase of stroke incidence in the young appears to be predominantly attributable to cryptogenic ischemic stroke (CIS) [2], implying there are unknown mechanisms that contribute to the etiology of young strokes. Some of these mechanisms may contribute to increased stroke risk in a very acute manner and these are termed ‘triggers’. The concept of triggers playing a role in acute stroke is well established [3].

Caffeine is the world’s most popular stimulant of which coffee, tea, soft drinks, and chocolate are among its most popular sources [4]. Caffeine is rapidly absorbed from the gastrointestinal tract and owing to no first pass metabolism by the liver, peak plasma concentration is reached in 15–120 min [5] and it is rapidly absorbed into the brain. The mechanism of action of caffeine is multi-faceted and includes antagonism of adenosine receptors (intracerebrally this is responsible for it increasing alertness) [6], inhibition of phosphodiesterase, calcium channel activation, and gamma-aminobutyric acid antagonism [5, 7].

Strong consensus supports case-crossover designs and analysis in the investigation of triggers. The Stroke Onset Study [8] explored non-alcoholic caffeinated beverage consumption as a trigger for ischemic stroke and found coffee was associated in the 1-h hazard period, even after adjustment for several variables. The ODYSSEY study [9], looking at a young (18–49 years) cohort, found an association with cola but not coffee in the 1-h hazard period for ischemic strokes. There were no associations seen with any caffeinated beverage in the CIS sub-group. To the best of our knowledge, there are no prior studies dedicated purely on investigating these triggers in CIS.

We investigated the role of acute non-alcoholic stimulant beverage consumption as a trigger for young CIS via a case-crossover analysis in a large, international, multi-center, case–control study.

## Methods

The Searching for Explanations for Cryptogenic Stroke in the Young: Revealing the Etiology, Triggers and Outcome (SECRETO; NCT01934725) study is a prospective case–control study of first ever young CIS patients. Between November 2013 and November 2022, patients aged 18–49 years were enrolled from 19 European centers. Ethical approval was obtained from the ethics committee of the hospital district of Helsinki and Uusimaa and by the local ethics committees of all participating sites. Written consent was obtained from all participants and in the instance where a patient did not have capacity, consent was obtained from a related proxy. Ethnicity was recorded for all study participants. National Institutes of Health Stroke Scale (NIHSS) score assessed stroke severity on admission.

The full study protocol has been previously published [10]. To be deemed eligible, patients had to have an acute diffusion-weighted imaging positive lesion on magnetic resonance (MR) imaging corresponding to neurological presentation, or a corresponding deficit on computed tomography (CT) perfusion scanning. For the stroke to be considered cryptogenic, patients underwent CT or MR angiography of extra- and intra-cranial vasculature (and any atherosclerotic disease had to be causing < 50% stenosis), a minimum of 24 h cardiac monitoring, standardized transesophageal and/or transthoracic echocardiography combined with bubble study and thrombophilia screening as per protocol [11]. All investigations had to be completed with 14 days of the presenting stroke. Cryptogenic was defined using the ASCO classification, with grades 0, II, and III (absence of disease, causality uncertain, or unlikely a direct cause) considered appropriate to classify as cryptogenic [12].

### Non-alcoholic caffeinated beverage consumption

Patients were asked about their consumption of coffee, tea, and cola using a structured questionnaire that was applied as soon as consent was secured. For coffee and tea, they were asked if they had consumed one cup of caffeinated coffee/tea in the last 12 months, how many cups they would consume on a typical day in the last year prior to the stroke, if they had consumed any coffee/tea in the 24 h preceding the stroke, and when the last cup of coffee/tea was consumed prior to the stroke. For cola, the same questions were asked but in relation to a standard can or bottle [8, 13].

### Vascular risk factors, stress, and patent foramen ovale

Vascular risk factors were identified from medical records and also through a structured questionnaire and were defined as the following: cardiovascular disease (any history of coronary artery disease, myocardial infarction, peripheral arterial disease or heart failure); hypertension (history of or current antihypertensive medication use, or mean systolic blood ≥ 140 mmHg or mean diastolic blood pressure ≥ 90 mmHg at study visit); diabetes mellitus (history of or current antidiabetic medication use); hypercholesterolemia (history of or current lowering medication use); abdominal obesity (waist–hip ratio > 0.85 in women and > 0.90 in men); physical inactivity (defined as not meeting the criteria for moderate or high levels of activity on the International Physical Activity Questionnaire) [14], current tobacco smoking (smoking at least one cigarette per day on average) and heavy alcohol consumption defined by > 7 units per week for women and > 14 units per week for men, or at least an average of 2 times per month and > 5 units for women and > 7 units for men per instance (binge drinking) were recorded [15]. Assessment of stress was performed using a modified version of the Perceived Stress Scale (PSS) [16, 17], with 10 questions looking at self-perceived stress over the month preceding the presenting stroke. They were categorized into 3 groups based on the total score: low (0–13), moderate (14–26), and high (27–40) stress.

Patent foramen ovale (PFO) was assessed via transthoracic or transesophageal echocardiography or transcranial Doppler, all with a bubble study and with documentation of high-risk features (atrial septal aneurysm and/or a large shunt).

### Statistical analysis

Categorical data for patients will be presented as frequencies (%) and continuous data as means with standard deviation (SD) or medians with interquartile range (IQR). Baseline characteristics are compared between the sexes using chi-squared test for categorical variables, and independent ‘*t*’ test for continuous variables if followed normal distribution, otherwise Wilcoxon rank-sum test if not following the normality assumption.

In a case-crossover analysis, there is a comparison of a pre-defined hazard period prior to stroke onset (case) and the period before this (control). As such, each patient serves as their own control. First, hazard values were calculated to produce a series of adjusted 2 × 2 tables with values weighted for exposure (available on request). Case-crossover analysis using these weighted exposures in the control (usual consumption in the preceding 12 months) and hazard period were performed using the Mantel–Haenszel method [18] to produce a relative risk (RR) of stroke for exposure in the hazard period with accompanying 95% confidence intervals (CI). This was performed for coffee, tea, and cola consumption at hazard periods of 1-h and 2-h pre-stroke. This analysis was then repeated by sex (male/female), age (18–39 years and 40–49 years), high-risk PFO status (yes/no), PSS score (low, moderate, and high stress levels) and traditional vascular risk factor burden (< 4 considered ‘low’ and ≥ 4 or more considered ‘high’ burden) as it was felt these were either substrates for ischemic stroke that may have been exacerbated by acute caffeine ingestion or psychological states that may have affected caffeine intake.

We also performed analysis according to NIHSS (0–6 versus more than 6), cognition measured by the Mini-Mental State Examination (MMSE, a score of 24 or more equating to normal) and the presence of aphasia at baseline to assess the possibility of recall being impacted by stroke severity or impairments in cognition or communication.

A further exploratory analysis was performed using the same triggers and hazard periods, but according to usual daily consumption of coffee and tea (≤ 2 cups versus ≥ 3 cups per day) and cola (≤ 2 cans versus ≥ 3 cans per day).

As a sensitivity analysis, conditional logistic regression was performed using exposure as a binary yes/no variable and comparing exposure between the hazard and control periods and reported as odds ratios (OR) with 95% CIs and p values. Sub-group analysis was then performed with the same variables as the Mantel–Haenszel method. During analysis, we found there was only enough statistical power to report on the 2-h hazard period.

Analysis was performed using RStudio (2024.12.1 + 563) and Stata version 19.5 (StataCorp., College Station, TX).

## Results

In total, 598 first ever cryptogenic ischemic strokes were analyzed. Mean age was 39.4 (SD 8.2) years. Of these, 54.7% (*n* = 327) patients were males, 94.1% (*n* = 563) were of white European ethnicity, and median NIHSS score was 2 (IQR 1–5).

Baseline characteristics of patients and univariate analysis by sex are presented in Table 1. Distribution of age (40.4 [SD 7.7] vs. 38.1 [SD 8.6], *p* < 0.001), hypercholesterolemia (5.2% vs. 1.2%, *p* = 0.03), abdominal obesity (73.2% vs. 43.5%, *p* < 0.001), smoking (35.9% vs. 26.8%, *p* = 0.02), and mean PSS score (11.7 [SD 6.7] vs. 15.1 [SD 7.4], *p* < 0.001) were significantly different between males and females.

**Table 1:** Baseline characteristics of patients in whole cohort and by sex

Variables (number of patients with missing data)	Whole Cohort (598)	Male (327)	Female (271)	*p* value
Age (mean, SD) (years)	39.4 (8.2)	40.4 (7.7)	38.1 (8.6)	** < 0.001**
Hypertension	223 (37.3)	131 (40.1)	92 (33.9)	0.12
Diabetes mellitus	23 (3.8)	15 (4.6)	8 (3.0)	0.30
Hypercholesterolemia (1)	22 (3.7)	17 (5.2)	5 (1.9)	**0.03**
Abdominal obesity (4)	355 (59.4)	238 (73.2)	117 (43.5)	** < 0.001**
Physical inactivity (7)	199 (33.3)	103 (32.0)	96 (35.7)	0.34
Smoking (3)	189 (31.6)	117 (35.9)	72 (26.8)	**0.02**
Heavy alcohol use (7)	76 (12.7)	45 (13.9)	31 (11.6)	0.39
Cardiovascular disease (1)	10 (1.7)	6 (1.8)	4 (1.5)	0.73
PSS score	13.3 (7.2)	11.7 (6.7)	15.1 (7.4)	** < 0.001**
High-risk PFO (25)	217 (37.9)	114 (36.3)	103 (39.8)	0.40

Data presented are means (SD) or number (percentages). Significant values in bold

*SD* standard deviation, *PSS* perceived stress scale, *PFO* patent foramen ovale

**Fig. 1:**
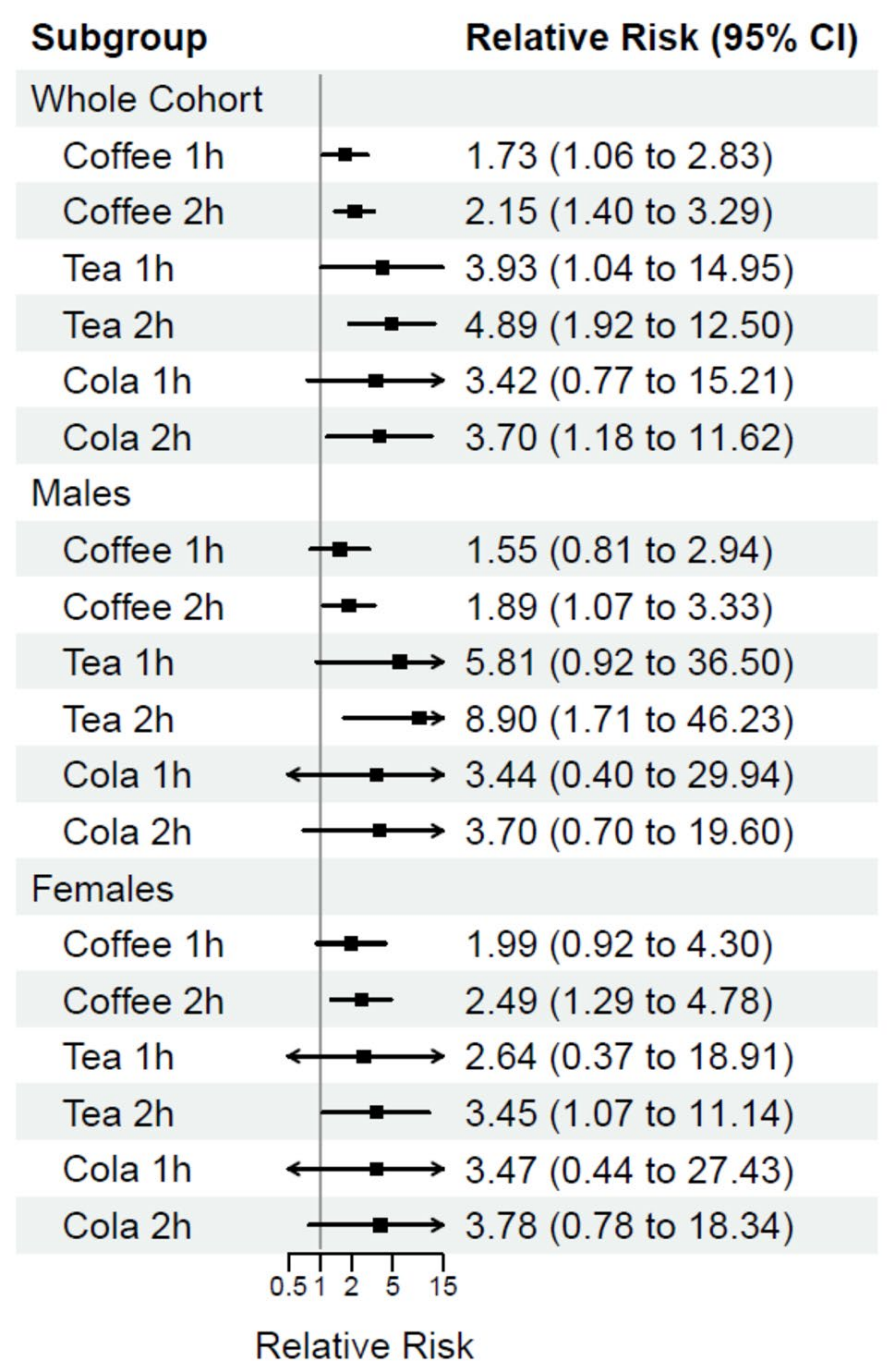
Forest plot showing case-crossover analysis using the Mantel–Haenszel method on the risk of developing cryptogenic ischemic stroke following coffee, tea, and cola exposure in 1-h and 2-h hazard periods for the whole cohort and by sex. Reported values are relative risks with 95% confidence intervals (CI)

Usual daily consumption of coffee, tea, and cola and their respective frequencies are shown in Table 2. Case-crossover analysis using the Mantel–Haenszel method of the whole cohort showed there was an association with the consumption of coffee in the 1-h (RR 1.73, 95% CI 1.06–2.83) and 2-h (2.15, 1.40–3.29) hazard periods, tea in the 1-h (3.93, 1.04–14.95) and 2-h (4.89, 1.92–12.50) hazard periods, and cola (3.70, 1.18–11.62) in the 2-h hazard period (Fig. 1).

**Table 2:** Usual daily consumption of coffee, tea and cola among the study population

	COFFEE	TEA	COLA
None	78 (13.1%)	220 (37.2%)	188 (31.9%)
Less than 2 cups/cans	184 (30.8%)	320 (54.1%)	364 (61.7%)
3–4 cups/cans	197 (33%)	32 (5.4%)	29 (4.9%)
5–6 cups/cans	90 (15.1%)	14 (2.4%)	4 (0.7%)
7–8 cups/cans	28 (4.7%)	2 (0.3%)	1 (0.2%)
9 or > cups/cans	20 (3.4%)	3 (0.5%)	4 (0.7%)
TOTAL	**597**	**591**	**590**

In exploratory sub-group analysis, when analyzed by sex, males had an association with coffee (1.89, 1.07–3.33) and tea (8.90, 1.71–46.23) consumption in the 2-h hazard period, and this was the same in females with coffee (2.49, 1.29–4.78) and tea (3.45, 1.07–11.14) (Fig. 1). The same associations in the 2-h hazard period were also seen in both age groups with coffee (2.32, 1.23–4.39) and tea (4.70, 1.28–17.25) in the 18–39-year age group and coffee (2.08, 1.17–3.72) and tea (5.05, 1.30–19.62) in the 40–49-year age group (Table 3).

**Table 3:** Case-crossover analysis using the Mantel–Haenszel method on the risk of developing cryptogenic ischemic stroke following coffee, tea, and cola exposure in 1-h and 2-h hazard periods by age. Reported values are relative risks with 95% confidence intervals

EXPOSURE	18–39 years	40–49 years
Coffee 1 h	1.88 (0.88–4.01)	1.65 (0.86–3.18)
Coffee 2 h	2.32 (1.23–4.39)	2.08 (1.17–3.72)
Tea 1 h	4.01 (0.58–27.76)	3.86 (0.61–24.42)
Tea 2 h	4.70 (1.28–17.25)	5.05 (1.30–19.62)
Cola 1 h	2.85 (0.45–17.95)	4.80 (0.35–65.63)
Cola 2 h	3.12 (0.76–12.79)	4.92 (0.66–36.41)

There was only an association with tea consumption (5.94, 1.84–19.20) in the 2-h hazard period in patients without a PFO, and with consumption of coffee (2.80, 1.44–5.43) in the 2-h hazard period in patients with a PFO. In both low- and high-risk factor burden sub-groups, coffee consumption in the 2-h hazard period was the only trigger to show an association (low: 1.96, 1.16–3.29 and high: 2.74, 1.27–5.89) (Table 4). Analysis by severity of the PSS score only showed an association with coffee consumption in the 2-h hazard period in the mild (2.41, 1.23–4.72) and moderate (2.76, 1.23–6.20) sub-groups (Table 5).

**Table 4:** Case-crossover analysis using the Mantel–Haenszel method on the risk of developing cryptogenic ischemic stroke following coffee, tea, and cola exposure in 1-h and 2-h hazard periods by patent foramen ovale (PFO) and vascular risk factor (VRF) burden status. Reported values are relative risks with 95% confidence intervals

EXPOSURE	No PFO	PFO	< 4 VRF	≥ 4 VRF
Coffee 1 h	1.48 (0.76—2.90)	2.08 (0.97—4.42)	1.63 (0.89–3.01)	1.99 (0.86–4.62)
Coffee 2 h	1.72 (0.97—3.05)	2.80 (1.44—5.43)	1.96 (1.16–3.29)	2.74 (1.27–5.89)
Tea 1 h	4.45 (0.95—20.85)	2.71 (0.17—42.75)	2.58 (0.24–27.71)	5.20 (0.99–27.30)
Tea 2 h	5.94 (1.84—19.20)	3.15 (0.53—18.63)	3.27 (0.81–13.28)	7.21 (1.96–26.52)
Cola 1 h	2.80 (0.39—19.86)	6.01 (0.48—75.15)	3.67 (0.55–24.54)	3.13 (0.28–35.16)
Cola 2 h	2.79 (0.67—11.61)	9.84 (0.94—103.08)	4.09 (0.94–17.72)	3.22 (0.51–20.17)

**Table 5:** Case-crossover analysis using the Mantel–Haenszel method on the risk of developing cryptogenic ischemic stroke following coffee, tea, and cola exposure in 1-h and 2-h hazard periods by Perceived Stress Scale (PSS) score categories. Reported values are relative risks (95% confidence intervals)

EXPOSURE	PSS (mild)	PSS (moderate)	PSS (severe)
Coffee 1 h	1.82 (0.86–3.84)	2.06 (0.83–5.16)	1.19 (0.12–12.14)
Coffee 2 h	2.41 (1.23–4.72)	2.76 (1.23–6.20)	1.28 (0.18–8.96)
Tea 1 h	2.84 (0.22–36.73)	3.32 (0.20–54.06)	3.78 (0.11–126.66)
Tea 2 h	2.89 (0.44–19.10)	4.43 (0.94–20.88)	5.57 (0.26–119.17)
Cola 1 h	3.26 (0.29–36.68)	2.73 (0.21–35.61)	n/a
Cola 2 h	2.93 (0.53–16.21)	3.24 (0.41–25.56)	n/a

n/a: numbers too small for analysis

Patients with and without aphasia on presentation showed an association with coffee and tea at the 2-h hazard period. Strokes with an NIHSS score of 0–6 showed an association with coffee and tea at both 1- and 2-h hazard periods, whereas a possible association was seen for cola in the 2-h hazard period for those with an NIHSS greater than 6 (Table 6). In those with a normal MMSE, both coffee and tea showed an association in 1- and 2-h hazard periods, but there were not enough patients with an MMSE below 24 to provide any meaningful statistical analysis.

**Table 6:** Case-crossover analysis using the Mantel–Haenszel method on the risk of developing cryptogenic ischemic stroke following coffee, tea, and cola exposure in 1-h and 2-h hazard periods by presence of aphasia and National Institutes of Health Stroke Scale (NIHSS) score. Reported values are relative risks (95% confidence intervals)

EXPOSURE	No Aphasia	Aphasia	NIHSS 0–6	NIHSS > 6
Coffee 1 h	1.69 (0.94–3.05)	1.79 (0.72–4.45)	1.69 (1.00–2.87)	2.01 (0.49–8.18)
Coffee 2 h	2.07 (1.25–3.42)	2.34 (1.04–5.26)	2.08 (1.32–3.28)	2.76 (0.77–9.93)
Tea 1 h	3.87 (0.90–16.58)	3.96 (0.13–119.91)	4.41 (1.06–18.39)	2.05 (0.04–99.37)
Tea 2 h	4.68 (1.51–14.51)	5.48 (1.02–29.45)	5.44 (1.90–15.59)	3.14 (0.39–25.51)
Cola 1 h	2.46 (0.35–17.23)	6.56 (0.33–129.19)	2.24 (0.27–18.71)	5.96 (0.63–56.65)
Cola 2 h	2.62 (0.61–11.22)	6.28 (0.62–63.17)	2.35 (0.49–11.22)	7.06 (1.11–44.90)

An additional exploratory analysis by usual daily consumption found an association with tea in the 2-h hazard period for those who drink ≤ 2 cups of coffee per day, with coffee in the 1- and 2-h hazard periods and cola in the 2-h hazard period in those who consumed ≤ 2 cups of tea per day and with both coffee and tea in the 1- and 2-h hazard periods in those who consume ≤ 2 cans of cola per day. There were no associations seen in any of the hazard periods for any beverage in those that consumed ≥ 3 cups of coffee or tea or ≥ 3 cans of cola per day (Table 7).In sensitivity analysis, conditional logistic regression of the 2-h hazard period found an association with consumption of coffee, tea, and cola. Exploratory analysis by sex found only coffee consumption in males and coffee and tea consumption in females were associated with CIS. Coffee consumption was associated with CIS in both age groups. Patients without a PFO showed association for both coffee and tea consumption and in those with a PFO, coffee only. Coffee consumption showed association in patients with mild and moderate PSS scores. Coffee also showed an association in patients with a low- and high-risk factor burden and NIHSS score 0–6 (Supplementary Table 1). In patients without aphasia, an association was seen with coffee and tea and in aphasic patients, with coffee only.

**Table 7:** Case-crossover analysis using the Mantel–Haenszel method on the risk of developing cryptogenic ischemic stroke following coffee, tea, and cola exposure in 1-h and 2-h hazard periods by usual daily consumption of coffee, tea and cola. Reported values are relative risks (95% confidence intervals)

EXPOSURE	COFFEE (≤ 2 cups)	COFFEE (≥ 3 cups)	TEA (≤ 2 cups)	TEA (≥ 3 cups)	COLA (≤ 2 cups)	COLA (≥ 3 cans)
1 h coffee	1.55 (0.31–7.62)	1.09 (0.64–1.87)	1.72 (1.03–2.84)	1.49 (0.14–15.88)	1.69 (1.02–2.82)	2.39 (0.36–15.85)
2 h coffee	1.61 (0.49–5.28)	1.17 (0.70–1.94)	2.14 (1.38–3.32)	1.64 (0.24–11.08)	2.07 (1.34–3.22)	4.26 (0.61–29.75)
1 h tea	4.01 (0.82–19.63)	3.28 (0.26–41.99)	1.81 (0.09–37.43)	1.23 (0.24–6.36)	3.96 (1.04–15.10)	n/a
2 h tea	4.87 (1.54–15.36)	4.40 (0.83–23.36)	1.90 (0.24–15.06)	0.99 (0.28–3.54)	4.94 (1.93–12.65)	n/a
1 h cola	3.50 (0.44–27.78)	3.44 (0.40–29.86)	4.01 (0.82–19.52)	1.19 (0.01–155.56)	1.62 (0.12–21.07)	1.85 (0.21–5.96)
2 h cola	3.47 (0.77–15.64)	4.38 (0.73–26.37)	4.34 (1.28–14.77)	1.22 (0.03–46.09)	1.69 (0.31–9.22)	3.68 (0.27–50.25)

n/a: numbers too small for analysis

When adjusted for the other non-alcoholic caffeinated beverages, coffee and tea consumption showed an association in the whole cohort, with coffee only in males and coffee and tea showing an association in females. Coffee consumption was associated with CIS in both age groups, and tea in the 40–49-year age group. Coffee was associated irrespective of PFO status and tea in those without a PFO. Coffee was an associated factor in mild and moderate PSS scores, in those with a low- or high-risk factor burden and irrespective of aphasia status, while tea was associated in patients without aphasia (Supplementary Table 2).

## Discussion

In this case-crossover analysis of young patients with CIS, we found that in the whole cohort, coffee, tea, and cola consumption were all potential trigger factors for young CIS within the 2-h hazard period. Coffee maintained this association in males, females, both age groups, PFO, mild and moderate PSS scores and low and high vascular risk factor burden groups, low NIHSS scores and irrespective of the presence of aphasia. Tea maintained this association in males, females, both age groups, no PFO, high vascular risk factor burden sub-groups, low NIHSS scores, and again irrespective of aphasia status. Recent cola consumption did not maintain any association on sub-group analysis. Interestingly, exploratory analysis suggested that there were no associations seen with any beverage in any hazard period in those who routinely consumed higher levels of non-alcoholic caffeinated beverages.

Several prior studies have assessed the role of triggers in acute stroke. An Israeli study [19] examined triggers in 200 ischemic strokes within a 2-h hazard period, comparing with that period 24 h earlier. This study did not specifically look at non-alcoholic caffeinated beverages as a trigger or specifically at young stroke patients but was one of the first studies to look in detail at the concept of triggers in stroke. In the case-crossover Stroke Onset Study [8], 390 ischemic strokes of all ages were interviewed a median of 3 days after stroke on recent coffee, tea, and cola consumption and consumption in the last year. In the 1-h hazard period, the RR for coffee consumption was 2.0 (1.4–2.8), with tea 0.9 (0.4–2.0) and cola 1.0 (0.4–2.4). The RR for coffee did not vary either by age, sex, smoking status, physical activity, or stroke etiology. When stratified by daily intake, it was only apparent in those consuming 1 or fewer cups per day in the last week and not by more regular consumers. The increased risk also returned to baseline at 2 h.

In the ODYSSEY study [9], patients aged 18–49-year-old with a first ever ischemic or hemorrhagic stroke completed a structured questionnaire on triggers and to date is the largest study to do so in this age group. 1-h and 2-h hazard periods for coffee and cola consumption were analyzed using the Mantel–Haenszel method. For any stroke, the risk increased for the 1-h hazard period for cola consumption (RR 2.0, 1.5–2.8) but not coffee (1.0, 0.8–1.2). When usual frequency was reduced from 24 to 16 h, RRs were lower. For ischemic stroke, cola consumption (1.9, 1.4–2.7) was again associated with an increased risk, but not coffee (1.0, 0.8–1.2). Stratified by Trial of Org 10,172 in Acute Stroke Treatment (TOAST) classification, cola consumption showed an increased RR in cardioembolic (2.8, 1.2–6.8) and other determined etiology (4.9, 2.6–9.3) with coffee not showing any association in any of the TOAST sub-groups. An analysis of patients with PFO-related young stroke did not find any association of coffee or cola consumption regardless of whether the PFO was associated with (coffee 0.92, 0.48–1.77, cola 2.08, 0.86–5.02) or a bystander (coffee 0.92, 0.35–2.44, cola 1.23, 0.12–12.75) to the stroke [20]. In our study which analyzed purely young CIS cases, we found coffee and tea consumption showed association in both the 1- and 2-h hazard periods and cola in the 2-h hazard period in the Mantel–Haenszel analysis of the whole cohort. Coffee also showed an association in patients with a PFO in the 2-h hazard period and tea in those without a PFO. However, it remains uncertain as to the role and mechanism of PFOs in this context and requires further studies. To our knowledge, our study is the largest assessment of these triggers in young CIS patients and provides evidence to suggest at some currently labeled cryptogenic strokes may have an acute trigger as a precipitant.

Consumption of up to 400 mg per day of caffeine is considered safe for adults [21, 22]. There is, however, distinct individual variability at absorption, metabolism, pharmacokinetic, pharmacodynamic, and receptor levels, which accounts for the variability in both plasma concentration and clinical effects of caffeine, in turn affecting future consumption [23, 24]. Other commonly used substances can affect the rate of caffeine clearance, with alcohol increasing the half-life and smoking decreasing it [25]. A 200 ml cup of coffee, 250 ml tea, and 355 ml cola contain approximately 90 mg, 28 mg, and 37 mg of caffeine, respectively, with some variations between specific brands [26, 27]. While long-term caffeine (in particular coffee) use has been implicated in positive health outcomes [21], vascular risk may arise from the direct effects of caffeine via acute sympathetic nervous system activation causing vasoconstriction and elevation in blood pressure with acute stress and changes on vascular endothelium [25]. However, a randomized controlled trial observing the cardiac effects of coffee consumption found no difference in premature atrial ectopics [28]. Caffeine also has a diuretic effect through inhibition of sodium and water in renal tubules [25], potentially having a dehydrating effect and increasing thrombosis. Indirectly, caffeine may act as a “trigger”, activating low risk, dormant or unknown pathologies that themselves pose an increased stroke risk. It has also been suggested tolerance is a phenomenon observed in habitual caffeine consumers and the above potential risks are more applicable to new or occasional consumers [8], which may be further compounded by the individual variation in caffeine handling. In our exploratory analysis, we observed associations only in those who routinely consumed lower levels of non-alcoholic caffeinated beverage, suggesting this is an important factor for consideration and requires validation in further studies.

Statistical methods used for case-crossover setting mandates a few considerations. The associations seen in the Mantel–Haenszel method and the conditional logistic regression sensitivity analysis observe similar trends though the conditional logistic regression analysis appears to show these trends to be much stronger. While the conditional logistic regression allows for the adjustment of other factors, we believe our analysis shows the importance of considering the weighting of an exposure to a trigger to obtain a more accurate assessment of the true risk of that trigger. This may also explain the much wider CIs in the associations seen in the conditional logistic regression model.

Our study had several strengths, being a large study exploring triggers of young CIS patients. The case-crossover design meant that patients were their own controls and thus this minimized selection bias of controls as risk factor profiles between cases and controls were identical. The structured questionnaire obtained detailed questions on non-alcoholic caffeinated beverage consumption allowing analysis using the Mantel–Haenszel method, which weighted the degree of caffeine consumption in the hazard and control period, thus allowing for a more accurate calculation of the relative risk. All patients were recruited and structured questionnaires completed within 14 days of stroke onset, thus minimizing recall bias and we performed sub-group analysis by stroke severity, MMSE scores, and aphasia status to see if these had an impact on any association, which they did not. However, recall bias remains a limitation in any retrospective questioning. There were also other limitations to this study. The study population was almost entirely of white European ethnicity and thus these results cannot be extrapolated to other ethnic groups. The conditional logistic regression results should be interpreted cautiously given that using a binary exposure eliminates the weighting of any small usual exposure and thus may falsely inflate the OR and widen the CI. Furthermore, we lacked certain details on beverage consumption, such as doses consumed within the 24 h preceding stroke. We also did not collect information on consumption of decaffeinated non-alcoholic beverages. Risk factors were defined using information from multiple sources and prevalence of some may have been underestimated; however, previous evidence has shown that e.g., hyperlipidemia and diabetes do not necessarily play a significant role in young CIS and will have had a limited impact on our findings [29].

## Conclusion

In this case-crossover analysis of 598 patients in the SECRETO study, we found that in the whole cohort, coffee, tea, and cola consumption were all potential trigger factors for young CIS within the 2-h hazard period, with coffee and tea maintaining a significant association in several sub-groups. Future studies should be focused on understanding the mechanism(s) behind these potential trigger factors.

## Supplementary Information

Below is the link to the electronic supplementary material.

Supplementary file1 (DOCX 20 KB)

## Data Availability

The authors report no relevant disclosures. Data are available upon direct request to the corresponding author and fulfillment of all statutory protocols.
