# Supplementary material for: Acute non-alcoholic caffeinated beverage consumption as a trigger for cryptogenic ischemic stroke in the young: findings from the SECRETO study
Source: J Neurol. 2026 Jun 11;273(7):388. doi: 10.1007/s00415-026-13882-2 (PMC13260097; doi:10.1007/s00415-026-13882-2)
Supplement: Supplementary file 1 — Supplementary file1 (DOCX 20 KB) [file 415_2026_13882_MOESM1_ESM.docx]

**Supplementary Table 1.**  Conditional logistic regression sensitivity analysis on the risk of developing cryptogenic ischemic stroke following coffee, tea, and cola exposure in the 2-hour hazard period in the whole cohort and by sex, age, PFO status, Perceived Stress Scale (PSS) score category and vascular risk factor (VRF) burden. Reported values are odds ratios with 95% confidence intervals.

|  | **COFFEE** | **TEA** | **COLA** |
| --- | --- | --- | --- |
| **Whole cohort** | 7.21 (4.13-12.62)* | 9.50 (2.21-40.78)* | 4.00 (1.13-14.17)* |
| **Males** | 6.33 (3.16-12.79)* | 6.00 (0.72-49.84) | 2.50 (0.49-12.89) |
| **Females** | 8.80 (3.49-22.19)* | 13.00 (1.70-99.37)* | 7.00 (0.86-56.89) |
| **18-39 years** | 11.75 (4.23-36.21)* | n/a | n/a |
| **40-49 years** | 5.40 (2.75-10.60)* | 4.50 (0.97-20.83)* | 1.33 (0.30-5.96) |
| **No PFO** | 5.09 (2.67-9.72)* | 6.00 (1.34-26.81)* | 4.00 (0.85-18.84) |
| **PFO** | 14.67 (4.55-47.23)* | n/a | 3.00 (0.31-28.84) |
| **PSS mild** | 6.67 (2.83-15.72)* | 2.5 (0.49-12.89) | 5.00 (0.58-42.80) |
| **PSS moderate** | 7.00 (2.46-19.96)* | n/a | 2.00 (0.37-10.92) |
| **PSS severe** | 7.00 (0.86-56.89) * | n/a | n/a |
| **<4 VRF** | 9.13 (4.40-18.93)* | 4.5 (0.97-20.83) | 7.00 (0.86-56.89) |
| **≥4 VRF** | 4.67 (1.93-11.27)* | n/a | 2.50 (0.49-12.89) |
| **No aphasia** | 6.55 (3.47-12.35)* | 6.50 (1.47-28.80) | 3.00 (0.81-11.08) |
| **Aphasia** | 9.67 (2.94-31.73)* | n/a | n/a |
| **NIHSS 0-6** | 8.27 (4.43-15.47)* | 15.00 (1.98-113.56) | 4.00 (0.85-18.84) |
| **NIHSS >6** | 3.33 (0.92-12.11) | 4.00 (0.45-35.79) | 4.00 (0.45-35.79) |

*p-value <0.05. n/a - values too small for analysis. PFO = patent foramen ovale.

|  | **COFFEE** | **TEA** | **COLA** |
| --- | --- | --- | --- |
| **Whole cohort** | 7.04 (3.91-12.68)* | 9.52 (2.09-43.46)* | 2.84 (0.77-10.50) |
| **Males** | 6.68 (3.12-14.30)* | 8.12 (0.81-80.86) | 1.72 (0.31-9.54) |
| **Females** | 7.63 (3.00-19.37)* | 10.38 (1.34-80.64)* | 5.13 (0.60-43.55) |
| **18-39 years** | 13.66 (4.23-44.12)* | n/a | n/a |
| **40-49 years** | 4.95 (2.48-9.86)* | 5.15 (1.05-25.34)* | 0.81 (0.16-4.19) |
| **No PFO** | 5.12 (2.58-10.19)* | 6.17 (1.29-29.43)* | 2.77 (0.56-13.75) |
| **PFO** | 12.67 (3.91-41.04)* | n/a | 3.00 (0.31-28.84) |
| **PSS mild** | 6.05 (2.54-14.39)* | 1.73 (0.24-12.51) | 2.56 (0.27-24.65) |
| **PSS moderate** | 6.25 (2.18-17.96)* | n/a | 2.00 (0.37-10.92) |
| **PSS severe** | 7.00 (0.86-56.88) | n/a | n/a |
| **<4 VRF** | 8.07 (3.85-16.90)* | 3.87 (0.68-21.92) | 4.49 (0.52-38.70) |
| **≥4 VRF** | 5.10 (1.96-13.31)* | n/a | 2.13 (0.40-11.31) |
| **No aphasia** | 5.90 (3.09-11.27)* | 6.18 (1.28-29.81) | 1.91 (0.48-7.57) |
| **Aphasia** | 13.00 (3.09-54.76)* | n/a | n/a |
| **NIHSS 0-6** | 7.72 (4.06-14.68) | 17.15 (2.08-141.63) | 2.71 (0.54-13.53) |
| **NIHSS >6** | 4.06 (0.87-19.06) | 3.10 (0.33-29.36) | 3.10 (0.33-29.36) |

**Supplementary Table 2.**  Conditional logistic regression sensitivity analysis on the risk of developing cryptogenic ischemic stroke following coffee, tea, and cola exposure in the 2-hour hazard period, adjusted for the other non-alcoholic caffeinated beverages in the whole cohort and by sex, age, PFO status, Perceived Stress Scale (PSS) score category and vascular risk factor (VRF) burden. Reported values are odds ratios with 95% confidence intervals.

*p-value <0.05. n/a - values too small for analysis. PFO = patent foramen ovale.
